# Efficacy of Antiviral Treatment in Hepatitis C Virus (HCV)-Driven Monoclonal Gammopathies Including Myeloma

**DOI:** 10.3389/fimmu.2021.797209

**Published:** 2022-01-11

**Authors:** Alba Rodríguez-García, María Linares, María Luz Morales, Sophie Allain-Maillet, Nicolas Mennesson, Ricardo Sanchez, Rafael Alonso, Alejandra Leivas, Alfredo Pérez-Rivilla, Edith Bigot-Corbel, Sylvie Hermouet, Joaquín Martínez-López

**Affiliations:** ^1^ Department of Translational Hematology, Instituto de Investigación Hospital 12 de Octubre (imas12), Hematological Malignancies Clinical Research Unit H120-CNIO, CIBERONC, Madrid, Spain; ^2^ Department of Biochemistry and Molecular Biology, Pharmacy School, Universidad Complutense de Madrid, Madrid, Spain; ^3^ Université de Nantes, Institut National de la Santé et de la Recherche Médicale (Inserm), Centre de Recherche en Cancérologie et Immunologie Nantes-Angers (CRCINA), Nantes, France; ^4^ Department of Microbiology, Hospital Universitario 12 de Octubre, Madrid, Spain; ^5^ Laboratoire de Biochimie, Centre Hospitalier Universitaire (CHU) de Nantes, Nantes, France; ^6^ Laboratoire d’Hématologie, Centre Hospitalier Universitaire (CHU) de Nantes, Nantes, France; ^7^ Department of Medicine, Medicine School, Universidad Complutense de Madrid, Madrid, Spain

**Keywords:** infection, hepatitis (C) virus, antiviral, multiple myeloma, monoclonal gammopathies

## Abstract

Multiple myeloma (MM) remains an incurable plasma cell malignancy. While its origin is enigmatic, an association with infectious pathogens including hepatitis C virus (HCV) has been suggested. Here we report nine patients with monoclonal gammopathy of undetermined significance (MGUS) or MM with previous HCV infection, six of whom received antiviral treatment. We studied the evolution of the gammopathy disease, according to anti-HCV treatment and antigen specificity of purified monoclonal immunoglobulin, determined using the INNO-LIA™ HCV Score assay, dot-blot assays, and a multiplex infectious antigen microarray. The monoclonal immunoglobulin from 6/9 patients reacted against HCV. Four of these patients received antiviral treatment and had a better evolution than untreated patients. Following antiviral treatment, one patient with MM in third relapse achieved complete remission with minimal residual disease negativity. For two patients who did not receive antiviral treatment, disease progressed. For the two patients whose monoclonal immunoglobulin did not react against HCV, antiviral treatment was not effective for MGUS or MM disease. Our results suggest a causal relationship between HCV infection and MGUS and MM progression. When HCV was eliminated, chronic antigen-stimulation disappeared, allowing control of clonal plasma cells. This opens new possibilities of treatment for MGUS and myeloma.

## Introduction

Multiple myeloma (MM) is a common hematologic malignancy (1.2% of all tumors) characterized by the clonal expansion and transformed plasma cells in the bone marrow. MM is always preceded by monoclonal gammopathy of undetermined significance (MGUS), an asymptomatic stage that does not always evolve to MM (1–3). Despite great advances in the understanding and treatment of MM, its origin is unknown, and it remains an incurable disease.

The primary function of plasma cells is to produce and secrete large amounts of immunoglobulins (Ig) that mediate humoral immunity against infection. Healthy plasma cells differentiate from immature B cells when they recognize an antigen foreign to the organism. This process occurs in the germinal centers of the secondary lymphoid organs, where B cells proliferate and select somatic hypermutations that have high affinity with the external antigen. In MM, monoclonal plasma cells secrete large quantities of a single Ig, monoclonal Ig, which serves as a marker of the disease and triggers much of the symptomatology (4).

Latent infection and chronic antigen stimulation are now recognized as initial pathogenic events leading to cancer. This association has been shown in several hematologic malignancies, such as chronic lymphocytic leukemia (CLL) and different types of lymphoma (5, 6). B-cell receptor (BCR) signaling is central for the specific recognition of Igs, suggesting that specific antigens could be involved in the development of different types of CLL. Interestingly, Hoogeboom and colleagues recently described a new subset of CLL that expresses stereotypic BCRs specific for β- (1, 6)-glucan, a major component of yeasts and fungi of the microbiota (7). The stimulation of BCR directed from these antigens seems to trigger signaling pathways through different mediators such as p53 and c-Myc, which result in proliferation, suppressed apoptosis, survival and alterations of cell migration (8).

In support of chronic antigenic stimulation as a pathogenic mechanism in MGUS and MM, several studies suggest an association between MM and viral infection, particularly hepatitis C virus (HCV), human immunodeficiency virus or Epstein Barr virus (EBV) (9–14). In addition, Nair et al. identified glucosylsphingosine (GlcSph) as the target of monoclonal Igs both in the context of Gaucher’s disease and in sporadic gammopathies (15, 16). Antigen-mediated stimulation led to an increase in the amount of monoclonal Ig and plasma cells in a murine model, confirming the role of chronic antigenic stimulation in the pathogenesis of MM. Independently, we recently reported that one-quarter of all MM cases might be initiated by infectious pathogens, including EBV and HCV (17, 18). In this line, a recent meta-analysis demonstrated a higher risk (2.67-fold) of developing MM in HCV-infected patients than in controls (11). These findings point to a role for HCV in the pathogenic development of MGUS and MM.

This concept opens new possibilities for treatment of MGUS and MM: target antigen reduction. If the target of the monoclonal Ig is eliminated, chronic antigen-stimulation disappears, leading to the control of clonal plasma cells. The efficacy of this therapeutic approach has been proven for GlcSph-associated MGUS and SMM (19).

In the present study, we explored the efficacy of anti-HCV treatment in a series of MGUS and MM patients linked to HCV. We report on a series of nine MGUS and MM patients with HCV infection, for whom the reactivity of the monoclonal Ig against HCV proteins was analyzed. We demonstrate for the first time that in cases where the monoclonal Ig reacted against HCV, treating the HCV infection improved MGUS and MM disease. Importantly, in a patient with refractory MM whose monoclonal IgG reacted specifically to HCV core protein, treatment of the HCV infection resulted in complete remission (CR) of MM, and the patient has been in clinically stable remission for four years.

## Materials and Methods

### Patients

Nine patients who developed MGUS (n=5) or MM (n=4) after HCV infection was detected in these patients were identified and classified into two groups: those who received antiviral treatment, and those who did not. The characteristics of patients at diagnosis are summarized in **Table 1**. Relevant information about the dates of diagnosis and treatments of the HCV infection and of the gammopathy are summarized in **Supplementary Tables S1, S2**. The study was approved by our Institutional Review Board and the patients provided written informed consent in accordance with the Declaration of Helsinki.

**Table 1:** Main patient characteristics at diagnosis of the gammopathy disease.

Patients	P1	P2	P3	P4	P5	P6	P7	P8	P9
Sex (M/F)	F	F	F	M	M	M	F	F	F
Age (years)	66	67	53	78	56	53	76	80	79
Diagnosis	MM IgGλ	MGUS IgGκ	MGUS IgGκ	MGUS IgAλ	MM Bence-Jones κ	MGUS IgAλ	MGUS IgGκ	MM IgAλ	MM IgGκ
Date of diagnosis	August 2011	January 2003	November 2017	June 2016	October 2014	September 2018	November 2015	January 2006	September 2016
Monoclonal Ig (g/dL)	5.67	1.72	0.74	1.47	0.0914	1.23	1.17	4.37	1.57
ISS	IIA	NA	NA	NA	III	NA	NA	IIA	III
Hemoglobin (g/dL)	12.8	15	15.1	12.6	10.3	15.9	10.5	11.6	10
Creatinine (mg/dL)	0.72	0.73	0.63	1.03	7.99	0.92	1.07	0.96	1.99
Bone Marrow Plasma Cells (%)	16	–	–	9	98	–	–	36	45
Platelets (10^9^/L)	163	170	309	173	190	104	206	152	231
Leukocytes (10^9^/L)	3.4	7.1	5	4.5	4.8	5.6	6.3	3.4	6.8
Calcemia (mmol/L)	2.4	2.5	2.3	2.4	2.3	2.3	2.4	2.5	2.3
β_2_-Microgobulin (mg/L)	2.9	–	2.1	–	13.07	–	–	6.44	5.9
High risk cytogenetics	No	–	–	–	No	No	Yes^a^	No	No
LDH high	No	No	Yes	No	Yes	No	No	No	Yes

M, male; F, female; Ig, immunoglobulin; MM, multiple myeloma; MGUS, monoclonal gammopathy of undetermined significance; ISS, Multiple Myeloma International Staging System; LDH, lactate dehydrogenase.

a High risk cytogenetics: del17p, t (4;14), t (14;16) or t (14;20).

NA, Not applicable.

### Immunofixation of Igs

Disease status was monitored by the quantification of monoclonal Ig or total Igs or free kappa/lambda light chain levels in serum, depending on the patient. Protein levels were routinely visualized by serum protein electrophoresis and/or immunofixation electrophoresis (20).

### Determination of Viral Load

Quantitative determination of RNA from HCV in human plasma containing K2EDTA was performed using the VERIS MDx system (Beckman Coulter). The RNA-HCV assay has been validated to provide quantitative results of samples containing HCV genotypes 1–6 (21). The main characteristics of the HCV infection in patients treated with antivirals are summarized in **Supplementary Tables S1, S2**.

### Purification of Monoclonal IgG and IgA

Agarose gel electrophoresis and purification of patients’ monoclonal Ig from other Igs present in serum samples was performed as described (17, 18, 22, 23) (**Figure S1** in the **Supplementary Appendix**). Protein concentrations were determined on a Nanodrop ND-1000 spectrophotometer. As an exception, in one patient diagnosed with Bence-Jones MM, kappa light chains were purified using PureProteomeTM Protein G and Kappa magnetic beads (Merck Millipore) and purity was evaluated by conventional native 15% polyacrylamide gel electrophoresis.

### Analysis of the Specificity of Antigenic Recognition of Purified Monoclonal Igs

The INNO-LIA™ HCV Score (Fujirebio) was used to analyze the reactivity of patient monoclonal IgG to HCV proteins. For monoclonal IgA, dot blotting assays with HCV proteins were performed on nitrocellulose membranes (Amersham) spotted with 1 μg of recombinant HCV core, NS3 and NS4 proteins (Abcam, Advanced Biotechnologies Inc.) (3 spots), which were then incubated with the patient’s serum or with the purified monoclonal IgA. The chemiluminescent microparticle immunoassay, Alinity i Anti-HCV (Abbott GmbH & Co. KG), on the Alinity i System was used for the qualitative detection of HCV in the patient diagnosed with Bence-Jones MM.

The multiplex infectious antigen microarray (MIAA) assay was used to analyze the reactivity of serum Igs and of purified monoclonal IgG or IgA against commercially available antigens and/or lysates from EBV, cytomegalovirus (CMV), herpes simplex virus-1 (HSV-1), herpes simplex virus-2 (HSV-2), varicella-zoster virus (VZV), *Helicobacter pylori (H. pylori)*, *Toxoplasma gondii*, and *Borrelia burgdorferi*, as described (14, 17).

## Results

### Absence of Disease Progression in Patients With HCV-Specific Monoclonal Ig Who Received HCV Antiviral Treatment

**Figure 1:**
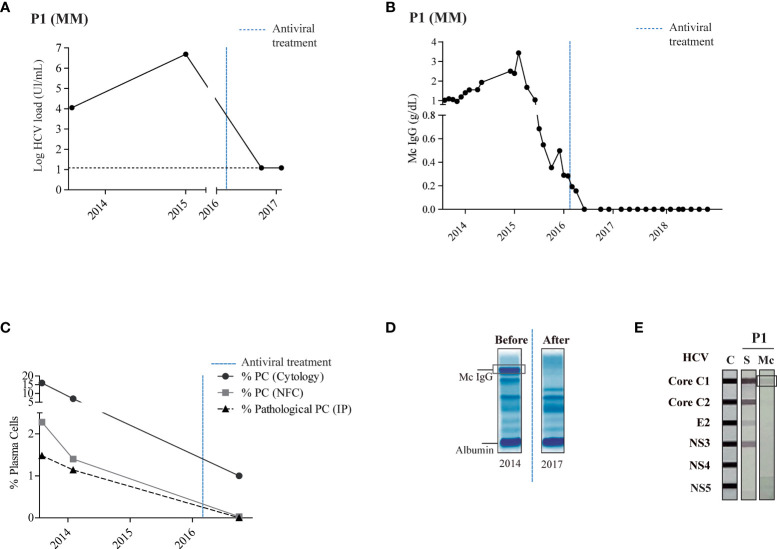
Stable complete remission and specific recognition of HCV in a patient with MM treated with antiviral drugs. Panel **(A)** shows the HCV load quantified by reverse-transcriptase quantitative polymerase chain reaction in the patient’s serum. The black horizontal dotted line represents the threshold of virus detection. Panel **(B)** shows the quantity of monoclonal IgG as determined by serum protein electrophoresis. Panel **(C)** shows the percentage of plasma cells determined by bone marrow examination (cytology) or next generation 8-color multiparametric flow cytometry, and the percentage of pathological plasma cells as determined by immunophenotyping. **(A–D)** The blue vertical dotted line indicates the time of antiviral treatment. Panel **(D)** shows a representative agarose gel electrophoresis of polyclonal Igs in serum before (2014) and after (2017) antiviral treatment – the encircled band corresponds to the patient’s monoclonal Ig. Panel **(E)** shows the INNO-LIA™ HCV test and immunoblotting assay used to detect reactivity of the patient’s serum IgG and of the purified monoclonal IgG against different HCV proteins. The signal obtained with HCV core for the monoclonal IgG of patient P1 was weak but always reproducible when different preparations of the purified monoclonal IgG were tested. C, positive controls; S, serum; Mc, monoclonal Ig.

Six patients had a monoclonal Ig that specifically recognized the HCV virus: patients P1–4, P7 and P8. Four of the patients (P1-4) received HCV antiviral treatment (detailed in **Table S1**). As expected, after antiviral treatment, HCV loads decreased, to undetectable levels for patients P1, P3 and P4 (**Supplementary Table S2**). For patient P1, who suffered from MM in third relapse at the time of anti-HCV treatment, eradication of HCV (**Figure 1A**) was associated with complete remission (CR) of MM in the absence of new anti-MM therapy. The patient’s monoclonal IgG drastically decreased (**Figure 1B**) and bone marrow aspirates showed < 5% of plasma cells by cytology and minimal residual disease negativity (**Figure 1C**). The number of plasma cell clones present in the sample and the tumor load was analyzed by next-generation sequencing, as previously reported (20, 24, 25). The monoclonal IgG present in pre-HCV treatment samples disappeared after anti-HCV treatment (**Figure 1D**). Forty-five months later, the patient remains in CR of MM with minimal residual disease negativity as assessed by next generation flow cytometry and undetectable monoclonal IgG. Patient P1’s purified monoclonal Ig specifically targeted the core protein of HCV (INNO-LIA™ HCV Score assay, **Figure 1E**).

**Figure 2:**
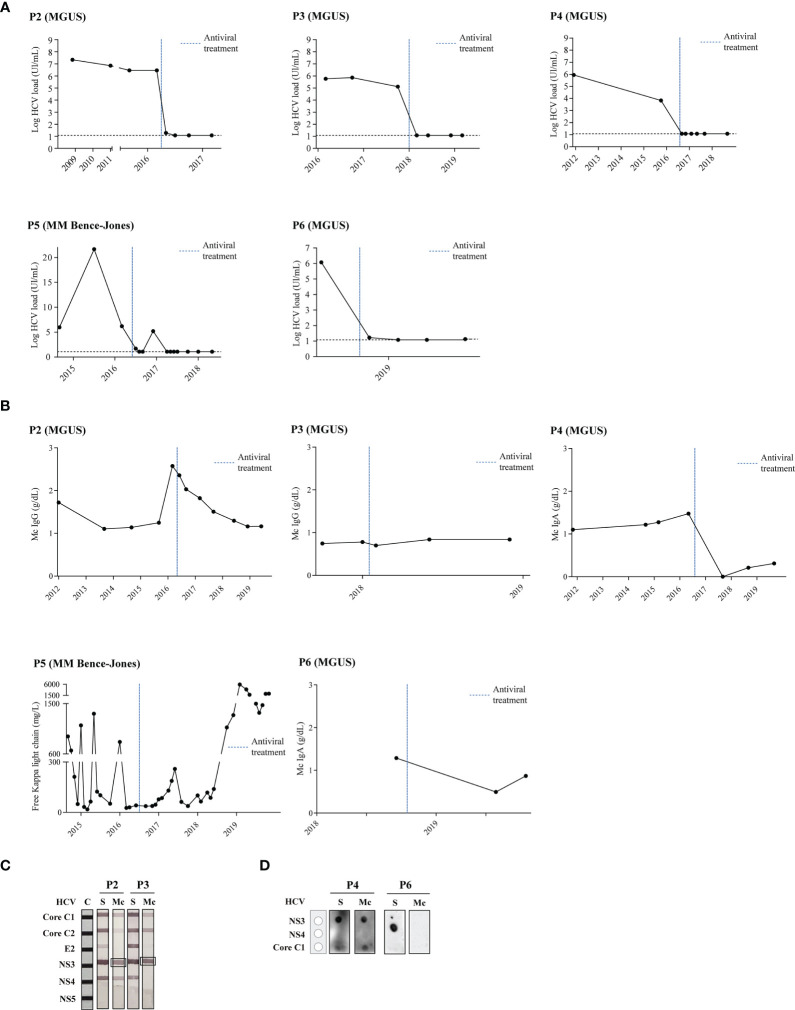
Disease evolution and evaluation of HCV-specificity of the monoclonal Ig from patients who were treated with antiviral drugs. Panel **(A)** shows the HCV load quantified by reverse-transcriptase quantitative polymerase chain reaction in patients’ serum. The black horizontal dotted line represents the threshold of virus detection. Panel **(B)** shows the quantity of monoclonal Ig as determined by serum protein electrophoresis. **(A, B)** The blue vertical dotted line indicates the time of antiviral treatment. Panel **(C)** shows the INNO-LIA™ HCV test to detect reactivity of patients’ serum IgGs and of the purified monoclonal IgG against different HCV proteins. Panel **(D)** shows the dot-blot assay to detect reactivity of patients’ serum IgAs and of the purified monoclonal IgA against different HCV proteins (IgAs cannot be studied using the INNO-LIA™ HCV test). The monoclonal IgA of patient P4 strongly recognized the HCV NS3 protein, whereas the monoclonal IgA of patient P6 did not recognize any HCV protein of the assay. C, positive controls; S, serum; Mc, monoclonal Ig.

When HCV-positive patients P2–4 (all MGUS patients) received antiviral treatment (and no other treatment), the HCV viral load decreased to minimum level in all cases (**Figure 2A** and **Supplementary Table S2**). Antiviral treatment was followed by a clear and stable decrease in the amount of monoclonal Ig decreased for patients P2 and P4. For patient P3, the concentration of monoclonal Ig decreased slightly and has now been stable for 39 months (**Figure 2B**). The purified monoclonal Ig from the aforementioned 3 MGUS patients (P2-4) specifically targeted antigens of HCV, either C1 (core) or NS3/NS4 proteins (**Figures 2C, D**). It should be noted that when dealing with MGUS, following the recommendations of the clinical guidelines, these patients did not receive any hematological treatment (**Supplementary Table S1**).

To confirm that the monoclonal Igs of patients P1-4 specifically recognized only HCV, we tested their reactivity against other microorganisms using the MIAA assay. Serological status (polyclonal Ig + monoclonal Ig) was evaluated in parallel. As expected, serum samples were reactive against several pathogens (EBV, CMV, HSV-1, HSV-2, VZV, *H. pylori, B. burgdorferi*, etc.). Confirming their purity and their specificity for HCV only, the monoclonal Ig preparations failed to react against any of these pathogens (**Figure S2** in the **Supplementary Appendix**).

### Disease Evolution in Patients for Whom the Monoclonal Ig’s Target Was Not Treated

Two patients with Bence-Jones (light chain) MM (P5) or MGUS (P6) were also successfully treated with antivirals (**Figure 2A** and **Supplementary Table S2**). However, their monoclonal Ig did not recognize HCV (**Figures 2C, D**) and these patients did not show an improvement of their MM (P5) or MGUS (P6) disease (**Figure 2B**). Patient P5 (light chain MM) presented two relapses after HCV therapy, despite different MM treatments (**Supplementary Table S1**). For patient (P6), light chain MGUS persisted with 7.5% plasma cells and a monoclonal component of 0.86g/dl 9 months after HCV therapy.

**Figure 3:**
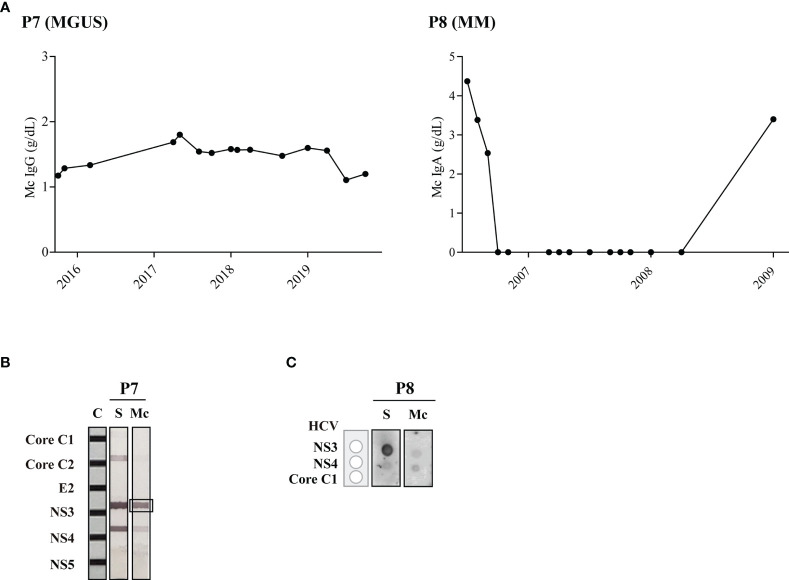
Disease evolution and evaluation of HCV recognition by the monoclonal Ig from patients who were not treated with antiviral drugs. Panel **(A)** shows the quantity of monoclonal IgG as determined by serum protein electrophoresis. Panel **(B)** shows the INNO-LIA™ HCV test used to detect reactivity of the serum IgGs and of the purified monoclonal IgG for Patient P7, which strongly recognized the HCV NS3 protein. Panel **(C)** shows the dot-blot assay used to detect reactivity of serum IgAs and of the purified monoclonal IgA in patient P8, which reacted against HCV NS3 protein. A weaker signal was noted for HCV NS4, possibly due to contaminating polyclonal IgAs. C, positive controls; S, serum; Mc, monoclonal Ig.

Three HCV-infected patients (P7-9) did not receive antiviral treatment, and disease evolution was unfavorable for all three patients. As shown in **Table 2**, within five months, MGUS patient P7 progressed to smoldering multiple myeloma (SMM) (17% of plasma cells). Despite various MM treatments (**Supplementary Table S1**), disease also progressed for patients P8 and P9, and both patients died. For patients P7 and P8, their purified monoclonal Ig specifically targeted the HCV NS3 protein (**Figure 3**), but for patient P9, the monoclonal Ig was shown to target a different virus, HSV-1.

**Table 2:** Classification of patients, response to anti-HCV treatment and MGUS or MM evolution.

Patients	Diagnosis	Monoclonal Ig	Purified	Target of the monoclonal Ig	Antiviral treatment	Decrease in monoclonal Ig	Disease progression	Length, progression-free (months)
P1	MM	IgGλ	Yes	HCV core C1	Yes	Yes	No, stable CR	45
P2	MGUS	IgGκ	Yes	HCV NS3	Yes	Yes	No	46
P3	MGUS	IgGκ	Yes	HCV NS3	Yes	Yes	No	26
P4	MGUS	IgAλ	Yes	HCV NS3	Yes	Yes	No	39
P5	MM Bence-Jones	Light chain κ	Yes	Unknown	Yes	No	Yes, 2 relapses	7
P6	MGUS	IgAλ	Yes	Unknown	Yes	No	No, stable MGUS	17
P7	MGUS	IgGκ	Yes	HCV NS3	No	No	Yes, to SMM	–
P8	MM	IgAλ	Yes	HCV NS3	No	No	Yes, death	–
P9	MM	IgGκ	Yes	HSV-1	No	No	Yes, death	–

MM, multiple myeloma; HCV, hepatitis C virus; CR, complete remission; MGUS, monoclonal gammopathy of undetermined significance; SMM, smoldering multiple myeloma; HSV-1, herpes simplex virus-1.

## Discussion

Previous reports have suggested an association between MGUS, MM and infection. In particular, we and others showed that at least one-third of MGUS and MM cases present with a monoclonal Ig that targets an infectious pathogen, including HCV (17, 22, 23, 26–28). The recent *in vivo* demonstration that chronic antigen stimulation can lead to emergence of clonal plasma cells supports the role of chronic infection in the development of subsets of MGUS and MM (15). This new pathogenic model is important since it offers, for the first time, the possibility of treatment for patients with MGUS, as well as novel therapeutic approach for MM: reduction or suppression of antigenic stimulation by treating the infection that initiated the gammapathy.

In support of this approach, the present study shows that whenever HCV-infected patients later diagnosed with MGUS or MM presented with a monoclonal Ig that targeted HCV, antiviral treatment markedly improved the outcome of their gammapathy. Importantly, we report for the first time that a long and stable CR has been achieved in a patient with refractory MM whose monoclonal IgG targeted HCV, after the sole administration of the antiviral treatment (with sofosbuvir and ledipasvir), and long after the administration of other hematological treatments. After 48 months, the patient remained in CR without MM symptoms. CR of MM is presumably linked to HCV disappearance since the antiviral drugs received by this patient have never been shown to have any anti-MM effect. For the other five HCV-positive MGUS cases, clonal HCV-specificity was also demonstrated through the recognition of HCV by the purified monoclonal IgG, and lack of recognition of other infectious pathogens, as assessed by the MIAA assay. Logically, for the two HCV-positive patients whose monoclonal Ig did not react against HCV, antiviral treatment had no favorable effect on the patient’s MGUS or MM disease (hereby confirming that anti-HCV drugs do not have intrinsic anti-MM effects).

In this study, we selected patients who had had HCV infection prior to the diagnosis of MGUS or MM. We were able to identify the target of the monoclonal Ig for 7/7 patients with a complete monoclonal Ig (heavy + light chains); the targets were HCV in 6 cases, HSV-1 in 1 case. These results are in line with our previous report that when MGUS or MM patients have a history of HCV infection, their monoclonal Ig target HCV in ~85% cases (9). Unfortunately, present assays do not allow to identify the target of clonal light chains, as observed for the two patients with light chain MGUS (P6) or MM (P5).

Thus, together with our previous studies, the present work demonstrates that it is feasible to identify the target of the monoclonal Ig of patients, then link the gammapathy to a previous chronic infection. This new approach should be useful for a significant fraction of MGUS and MM patients: for instance, a recent study revealed that until 7.4% of MM patients were positive for HCV RNA by RT-PCR assay. In this study, the authors emphasize that serologic tests at the time of diagnosis of MM are necessary to identify infected patients, and they propose that confirmation of positive cases by molecular techniques should be mandatory (29). Whether the gammapathy is linked to the infectious agent can then be addressed by showing that the patient’s monoclonal Ig reacts against the pathogen. This information allows to propose antiviral or antibiotic treatments to MGUS patients (presently not treated), with the aim of curing the MGUS, and also to SMM and MM patients, as adjuvant treatments, with the aim to improve the patient’s response to chemotherapy. This “target antigen reduction” approach should improve the prognosis in patients with gammopathies, especially those at the MGUS or SMM stages. In fact, previous studies reported the need for a complete follow-up of patients with chronic infection due to the possibility that they develop MM or non-Hodgkin lymphoma (NHL) (30). Panfilio et al. (31) previously reported a MM regression after antiviral treatment of one patient with HCV infection; unfortunately, the antigen targeted by the patient’s monoclonal Ig was not investigated by these authors (31). Similarly, the beneficial effect of interferon treatment against MM in HCV-infected patients was reported recently by Ioannou et al. (32). Regrettably, the antigen specificity of the monoclonal Igs of patients was not studied by these authors (32). In contrast, our study establishes that anti-HCV treatment improves and even suppresses MGUS or MM disease when the gammopathy is driven by HCV, i.e. when the patient’s monoclonal Ig targets HCV. Inversely, when the monoclonal Ig did not target HCV, anti-HCV treatment had no effect on the gammapathy, an indirect confirmation that the beneficial effect of antiviral therapy on MGUS and MM acts *via* the clearance of the HCV infection. Similar observations have been made for HIV-infected patients diagnosed with MM, for whom HIV treatment resulted in a significant reduction in serum monoclonal Ig, a superior response to MM therapy and improved overall and progression-free survival (33). Although the specificity of the monoclonal Ig of these patients was not studied, these observations were consistent with a beneficial role of antiretroviral therapy in terms of control of the plasmacytic clone and Ig production (33).

Despite the fact that HCV has long been shown to be a risk factor for the development of certain proliferative diseases, including MM, the underlying mechanisms remained unclear. Although not completely understood, the mechanisms of HCV cell entry involve the binding of the E2 protein of the virus envelope and CD81, a molecule which is very abundant on the surface of hepatocytes and also of B lymphocytes (34–36). Hence, HCV can infect both hepatocytes and B-cells, and thus directly induce genetic alterations in infected cells and the subsequent development of hepatic or/and B-cell malignancies. However, in the context of MGUS and MM, the most frequent mechanism of cell transformation by HCV is likely to be indirect, since HCV antigens are detected in peripheral mononuclear cells in chronically infected patients (37). Studies based on the importance of sustained stimulation over time by HCV antigens suggest a mechanism of action analogous to the one at play during infection by *H. pylori*, which increases the risk of indirect carcinogenesis and lymphomagenesis in patients infected with a virulent strain. *H. pylori* infection induces gastric inflammation and chronic antigen stimulation of B-cell immune responses, hereby facilitating the acquisition of genetic alterations and transformation of infected gastric tissues as well as cells of the B lineage. In infected patients, it is not rare to observe the presence of oligoclonal Igs then of a monoclonal Ig (thus of a plasmocytic clone), which normally disappear rapidly. The same process is likely at play in infection-initiated MGUS, except that the plasmacytic clone and monoclonal Ig persist for more than 6 months, often for several years, eventually progressing toward MM. Our data show that in the context of HCV infection, such clones remain antigen-dependent, since successful antiviral treatment results in the reduction or suppression of the plasmocytic clone and monoclonal Ig.

In summary, our study highlights the urgency of treating HCV in infected patients, especially in MGUS, SMM or MM cases who present a monoclonal Ig that reacts against the virus, prior to chemotherapy schemes. Importantly, antiviral treatment of all HCV-positive patients should prevent the development of HCV-driven MGUS and MM. Overall, our findings suggest that chronic stimulation by HCV may promote the development of MGUS and MM in chronically infected patients. This observation has evident clinical consequences, since the identification of a patient with a monoclonal Ig specific for a treatable pathogen, such as HCV, would possibly allow curative antiviral treatment in case of MGUS, and improved response to chemotherapy schemes in case of MM.

## Data Availability Statement

The original contributions presented in the study are included in the article/**Supplementary Material**. Further inquiries can be directed to the corresponding author.

## Ethics Statement

The studies involving human participants were reviewed and approved by Comité de Ética del Instituto de Investigación Hospital 12 de Octubre. The patients/participants provided their written informed consent to participate in this study.

## Author Contributions

AR-G and ML performed experiments, analyzed data, and wrote the initial manuscript draft. MLM contributed to analyzed and discuss the data. SA-M and NM performed experiments and analyzed data. RS and EB-C contributed to experiments and analyzed the data. RA, AL and AP-R contributed with patient samples and data. SH and JM-L designed the research, provided resources and financial support and edited the manuscript. All authors contributed to the article and approved the submitted version.

## Funding

This work was supported by grants to SH from the Ligue Nationale contre le Cancer (Comités Départementaux 44, 56, 29, 85, 35) and International Myeloma Foundation (IMF) (Brian D. Novis senior grant). We acknowledge the Instituto de Investigación Sanitaria Hospital 12 de Octubre (imas12), CIBERONC, AECC (Accelerator Award and Ideas Semilla), and the CRIS foundation for their help. MLM has a grant from the Spanish Society of Hematology.

## Conflict of Interest

The authors declare that the research was conducted in the absence of any commercial or financial relationships that could be construed as a potential conflict of interest.

## Publisher’s Note

All claims expressed in this article are solely those of the authors and do not necessarily represent those of their affiliated organizations, or those of the publisher, the editors and the reviewers. Any product that may be evaluated in this article, or claim that may be made by its manufacturer, is not guaranteed or endorsed by the publisher.
