# Supplementary material for: Efficacy of Antiviral Treatment in Hepatitis C Virus (HCV)-Driven Monoclonal Gammopathies Including Myeloma
Source: Front Immunol. 2022 Jan 11;12:797209. doi: 10.3389/fimmu.2021.797209 (PMC8786723; doi:10.3389/fimmu.2021.797209)
Supplement: Supplementary file 1 [file DataSheet_1.pdf]

Supplementary Material

This appendix has been provided by the authors to give readers additional information about their work.

Supplement to: Efficacy of antiviral treatment in Hepatitis C virus (HCV)-driven monoclonal gammopathies including myeloma

1 Supplementary Methods

Next-generation flow cytometry analysis

Bone marrow samples were immunophenotyped using next generation 8-color multiparametric flow cytometry as reported (1). During follow-up, immunophenotypic response was defined as the absence of detectable MM plasma cells by multiparametric flow cytometry at a sensitivity level of 10^{-5}, following International Myeloma Working Group criteria.

Next-generation sequencing

For patient P1, the number of plasma cell clones present in the sample and the tumor load were analyzed by next-generation sequencing. Standardized primers developed by the Biomed-2 concerted action to amplify Ig heavy (IgH) and lambda (Igλ) chain sequences were used, as described (2). The sequencing data were analyzed to identify and quantitate the clone-specific sequence (clonotype) present in each sample. A clonotype was identified when at least 400 identical sequencing reads were obtained or were present at a frequency >1%.

2 Supplementary Figures and Tables

Supplementary Table S1. Information about the dates of diagnosis and treatments of the HCV infection and of the gammopathy.

	Date of HCV detection	*Date of MGUS or MM diagnosis*	*Date of anti-viral treatment*	*Antiviral treatment*	*Date of haematological treatments*	*Haematological treatments*
P1	*December 1992*	*August 2011*	*March 2016*	*Sofosbuvir + Ledipasvir*	*September 2013*	*NK therapy+ Lenalidomide*
					June 2014	*Bortezomib+Lenalidomide +Dexamethasone*
					February 2015	*Bendamustine-Bortezomib-Dexamethasone*
P2	*Unknown (before 2003)*	*January 2003*	*may-16*	*Paritaprevir / Ritonavir-Ombitasvir + Dasabuvir*	*NA*	*Not treated (MGUS)*
P3	*January 2000*	*November 2017*	*January 2018*	*Glecaprevir + Pibrentasvir*	*NA*	*Not treated (MGUS)*
P4	*January 1996*	*June 2016*	*August 2016*	*Sofosbuvir + Ledipasvir*	*NA*	*Not treated (MGUS)*
P5	*Unknown (before 2014)*	*October 2014*	*July 2016*	*Sofosbuvir + Ledipasvir*	*October 2015 and October 2016*	*Lenalidomide+Dexamethasone*
					April 2017	*Clinical Trial: m14-031 VD+Venetoclax/Placebo*
P6	*February 1995*	*September 2018*	*October 2018*	*Glecaprevir + Pibrentasvir*	*NA*	*Not treated (MGUS)*
P7	*Before April 2017*	*November 2015*	*NA*	*Not treated*	*NA*	*Not treated (MGUS)*
P8	*Before September 1978*	*January 2016*	*NA*	*Not treated*	*July 2006*	*Clinical trial GEM05: Melfalan, Bortezomib and Prednisona*
					April 2009	*Lenalidomide+Dexamethasone*

P9	*Unknown*	*September 2016*	*NA*	*Not treated*	*June 2016*	*EC TCD13983 Isatuximab, Velcade and Ciclofosfamida (NCT02513186)*
					November 2016	*Lenalidomide+Dexamethasone*

Supplementary Table S2. Main characteristics of HCV infection.

Patients	HCV serotype	Viral Load[a] (Log) before the treatment	Viral Load[a] (Log) after the treatment
P1	1b	6.69	1
P2	1b	6.46	1.27
P3	4	5.10	1
P4	1b	3.83	1
P5	1a	6.19	1.68
P6	3	6.07	1.23

Abbreviations: HCV, hepatitis C virus. [a] Note: The detection limit of the viral load determination technique is 1.

Supplementary Figures

Supplementary Figure 1. Purification of monoclonal Igs and evaluation of purity by isoelectrofocusing and immunoblotting.

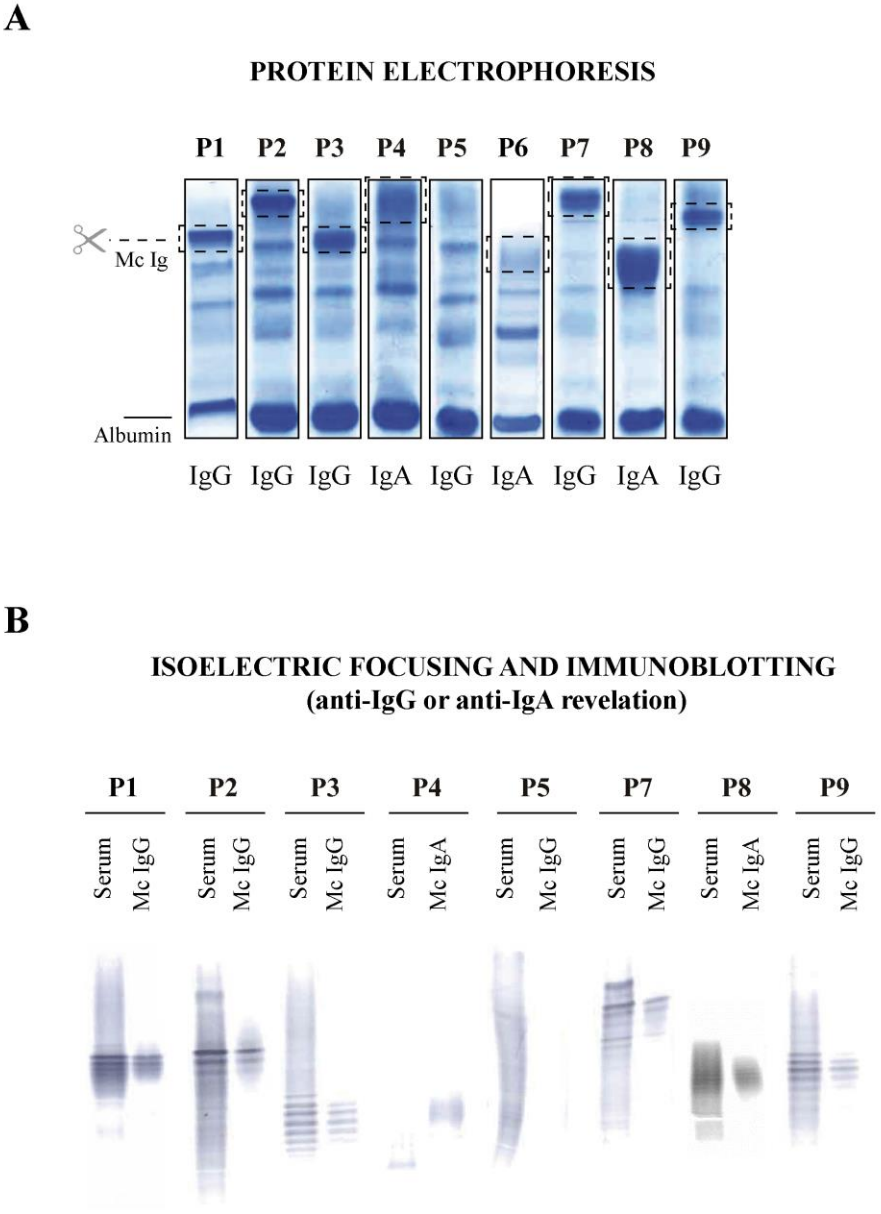

Supplementary Figure 1. After clotting, blood samples were centrifuged at 2200 × g (4°C) and serum aliquots were frozen. Measurement of Ig concentration, purification of monoclonal IgG and IgAs, and verification of the purity of monoclonal Ig preparations were performed as described (3–5). Briefly, Ig concentration in serum was measured with an immunonephelometric assay performed on a Beckman Image Analyzer (Beckman Coulter). (A) After protein separation using electric charge on agarose gel electrophoresis (SAS-MX high resolution, Helena Biosciences), the band corresponding

to the monoclonal Ig was carefully excised and proteins were eluted from gels into PBS. Concentration of the purified monoclonal Ig was determined using an ND-1000 Nanodrop Spectrophotometer. (B) Purity of each monoclonal Ig fraction was analyzed by isoelectrophoresis and immunoblotting (homemade isoelectrofocusing gel using a range of pH 3–10, blotting onto PVDF membranes and detection using a horseradish peroxidase (HRP)-conjugated rabbit anti-human IgG (Heavy + Light chains) for IgGs, and HRP-conjugated rabbit anti-human IgA alpha chain for IgAs. For patient P6, too little purified monoclonal Ig was obtained, was not visible on the IEF (not shown), and the target of the monoclonal Ig could not be determined (Table 2).

Supplementary Figure 2. Serological status against other pathogens assessed by MIAA assay

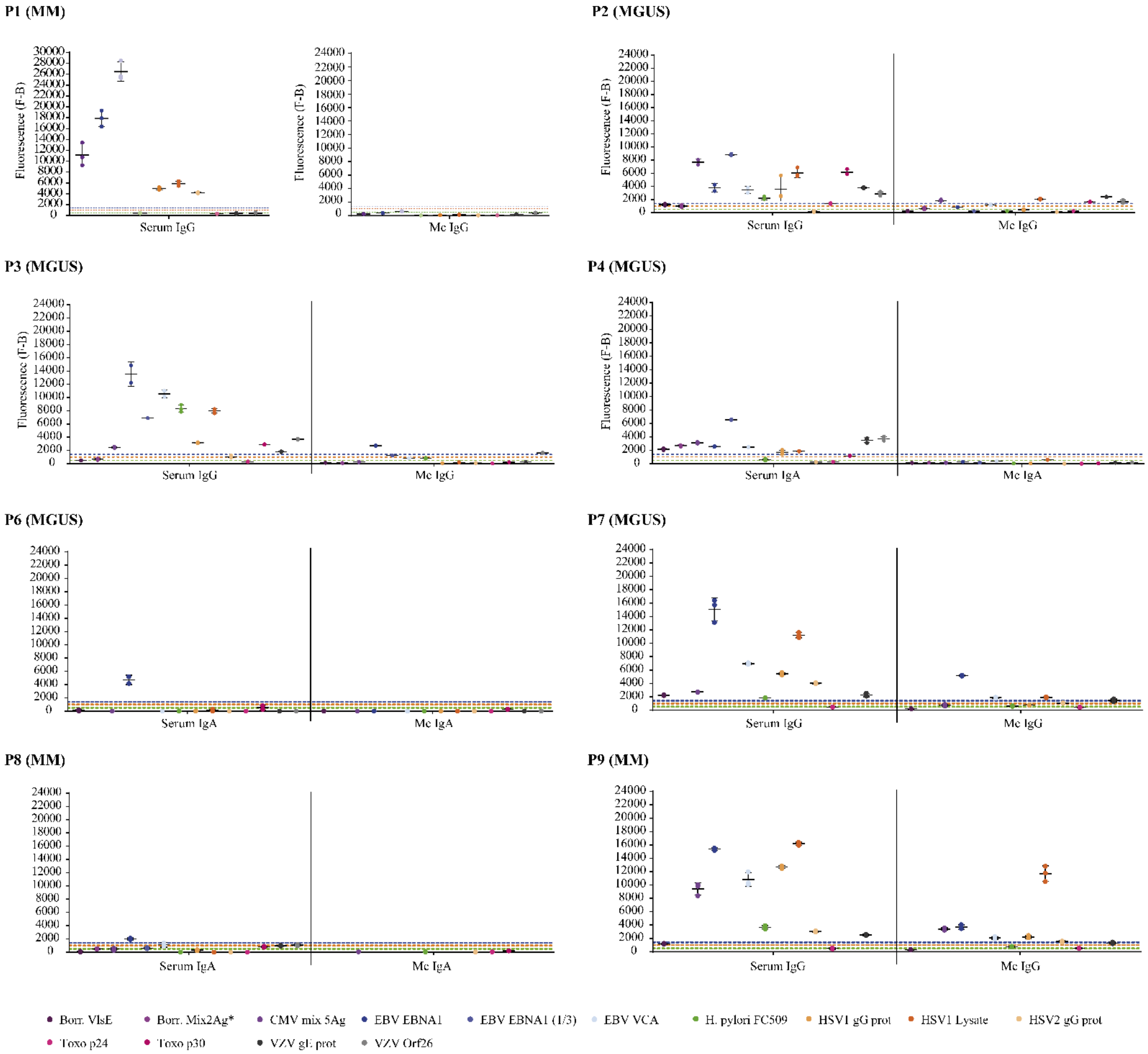

Supplementary Figure 2. The MIAA assay was used to analyse the reactivity of serum Ig (G or A) and of the purified monoclonal Igs (G and A) against 8 infectious pathogens: *Borrelia burgdorferi*:

VisE protein and a mix of two antigens); cytomegalovirus (CMV): a mix of five antigens; Epstein-Barr virus (EBV): nuclear antigen-1 (EBNA-1) and viral capsid protein (VCA); *Helicobacter pylori*: FC509; Herpes simplex virus-1 (HSV-1): gGprotein and a lysate; Herpes simplex virus-2 (HSV-2): gGprotein; *Toxoplasma gondii*: proteins 24 and 30; varicella-zoster virus (VZV): gEprotein and Orf26. For each patient, serum Igs (polyclonal Igs + monoclonal Ig) and the purified monoclonal Ig preparation were analysed in parallel. For hybridization, Ig concentrations were adjusted to 400 µg/mL for serum and from 50 to 200 µg/mL for purified monoclonal Ig. 80 µL of samples were incubated for 2 hours at room temperature. After washing, slides were incubated with a labelled secondary antibody (0.2 µg/ml DylightTM 680 labeled goat anti-human IgG (H+L), or anti-human IgA (SeraCare). Fluorescence signalling, detected with the Odyssey infrared imaging system scanner at 21 µm resolution (LI-COR Biosciences), was used to determine the serological status of each sample. Specific fluorescence was quantified using GenePix® Pro 4 Microarray Acquisition & Analysis Software (Molecular Devices)3–5. Three fluorescence thresholds of specific positivity were used in MIAA experiments: 500, for H. pylori, and *T. gondii* (green dotted line); 1000, for CMV, HSV-1, and HSV-2 (orange dotted line); and 1400, for EBV, VZV, and *B. burgdorferi* (blue dotted line). Signals below these thresholds are considered to be negative. Dots may be superimposed; black horizontal bars represent the means of results obtained for a pathogen, protein or lysate. Positive and negative controls are run in every MIAA experiment as internal controls.

For patients P1–4 and P7–8, the purified monoclonal Ig recognized HCV; accordingly, in contrast to the patient's serum, the MIAA showed no signal for monoclonal Ig of patients P1, P2, P4 and P8. The monoclonal Ig preparations of patients P3 and P7 gave weak signals for EBV EBNA-1 (compared with serum Igs), likely due to contaminating polyclonal Igs specific for EBV EBNA-1. For patient P6, too little purified monoclonal Ig was obtained, and no signal was evident with the MIAA. For patient P9, the purified monoclonal Ig specifically recognized HSV-1; other weak signals (compared with the signals obtained with serum Igs) were observed, likely explained by the presence of contaminating polyclonal Igs. For patient P5 (Bence-Jones MM), the MIAA could not be performed.

REFERENCES

1. Martinez-Lopez J, Lahuerta JJ, Pepin F, Gonzalez M, Barrio S, Ayala R, Puig N, Montalban MA, Paiva B, Weng L, et al. Prognostic value of deep sequencing method for minimal residual disease detection in multiple myeloma. Blood (2014) 123:3073–3079. doi:10.1182/blood-2014-01-550020

2. Martinez-Lopez J, Sanchez-Vega B, Barrio S, Cuenca I, Ruiz-Heredia Y, Alonso R, Rapado I, Marin C, Cedena M-T, Paiva B, et al. Analytical and clinical validation of a novel in-house deep-sequencing method for minimal residual disease monitoring in a phase II trial for multiple myeloma. Leukemia (2017) 31:1446–1449. doi:10.1038/leu.2017.58

3. Feron D, Charlier C, Gourain V, Garderet L, Coste-Burel M, Le Pape P, Weigel P, Jacques Y, Hermouet S, Bigot-Corbel E. Multiplexed infectious protein microarray immunoassay suitable for

the study of the specificity of monoclonal immunoglobulins. Anal Biochem (2013) 433:202–209. doi:10.1016/j.ab.2012.10.012

4. Bosseboeuf A, Feron D, Tallet A, Rossi C, Charlier C, Garderet L, Caillot D, Moreau P, Cardó-Vila M, Pasqualini R, et al. Monoclonal IgG in MGUS and multiple myeloma targets infectious pathogens. JCI Insight (2017) 2: doi:10.1172/jci.insight.95367

5. Bosseboeuf A, Allain-Maillet S, Mennesson N, Tallet A, Rossi C, Garderet L, Caillot D, Moreau P, Piver E, Girodon F, et al. Pro-inflammatory State in Monoclonal Gammopathy of Undetermined Significance and in Multiple Myeloma Is Characterized by Low Sialylation of Pathogen-Specific and Other Monoclonal Immunoglobulins. Front Immunol (2017) 8: doi:10.3389/fimmu.2017.01347
